# Adaptive speed control of BLDC motors for enhanced electric vehicle performance using fuzzy logic

**DOI:** 10.1038/s41598-025-90957-6

**Published:** 2025-04-12

**Authors:** R. Shenbagalakshmi, Shailendra Kumar Mittal, J. Subramaniyan, V. Vengatesan, D. Manikandan, Krishnaraj Ramaswamy

**Affiliations:** 1 Department of Electrical & Electronics Engineering, Vel Tech Multi Tech Dr.Rangarajan Dr.Sakunthala Engineering College, Avadi, Chennai, India; 2 Department of Electrical Engineering, G H Raisoni College of Engineering and Management, Pune, India; 3 Department of Electrical & Electronics Engineering, SRM TRP Engineering College, Tiruchirappalli, India; 4 Department of Mechanical Engineering, SRM TRP Engineering College, Tiruchirappalli, India; 5 Department of Mechanical Engineering, College of Engineering and Technology, Dambi Dollo University, Dambi Dollo, Ethiopia; 6 Center for global health research, Saveetha institute of medical and technical sciences, Saveetha University, Chennai, India

**Keywords:** BLDC motor, Electric vehicles, Fuzzy logic controller, Membership functions, PID controller, Propulsion system, Speed control, Engineering, Electrical and electronic engineering

## Abstract

This study investigates the use of a closed-loop speed control approach based on fuzzy logic for brushless DC (BLDC) motors in Electric Vehicle (EV) applications. The primary objective is to overcome the drawbacks of traditional control techniques by improving dynamic performance, response time, and stability under changing load conditions and parameter uncertainty. Nonlinearities, load fluctuations, and transient overshoots are common problems for traditional PID controllers, which results in suboptimal performance of EV propulsion systems. A state-space modelling technique for the BLDC motor is used in this study to address these issues, incorporating a Fuzzy Logic Controller (FLC) for accurate speed control. The superiority of FLC over PID controllers is demonstrated by a comparison study that was verified by simulation and hardware implementation. The results show that FLC produces smooth speed transitions, no overshoot, and zero steady-state error with a settling time of only 0.05s, as in contrast to 0.1s for the PID controller. Under load fluctuations, the FLC’s torque response stays constant at about 1.05 Nm, however the PID controller shows noticeable oscillations and a larger torque ripple. Additionally, FLC guarantees smooth speed regulation throughout a broad range (1500–3000 rpm), greatly increasing motor lifespan and energy efficiency. When compared to the PID controller, the experimental validation shows that FLC performs robustly in real-time EV settings, exhibiting smoother speed transitions, faster disturbance rejection, and improved adaptability. According to these results, FLC is a better option for BLDC motor speed control in EV applications, guaranteeing effective propulsion, less mechanical stress, and more driving stability. As a potential control strategy for upcoming EV technologies, the proposed strategy not only improves energy utilisation but also strengthens system reliability.

## Introduction

The growing popularity of electric vehicles (EVs) has created a dramatic change in the global energy landscape. In 2023, over 14 million electric vehicles were sold globally, and this year, sales are predicted to increase by another 35%^1^. It is forecasted that the share of the overall auto market accounted for by electric vehicles will increase from approximately 4% in 2020 to 14% in 2022, 18% in 2023 and is expected to reach 23.5% in 2025, then 45.3% in 2030, and 68.4% in 2035, due to their fast development^2^. Electric motor technologies, especially brushless DC (BLDC) motors, which are essential to EV propulsion systems, are advancing as a result of this change, which is also changing the automotive sector. Because of their exceptional efficiency, small size, and regenerative braking characteristics, BLDC motors have become the preferred choice for EV applications. BLDC motors provide lower mechanical complexity, greater energy efficiency, and zero tailpipe emissions in contrast to internal combustion engines^3^.

Because of their high power density, reliability, and accurate control, Permanent Magnet Brushless DC (PMBLDC) motors stand out among BLDC motors and are perfect for the dynamic needs of electric vehicles^4^. However, the BLDC motors lack mechanical commutators, they require complex electronic control systems, which come with drawbacks such as sensor dependence, electromagnetic interference (EMI), and expensive startup expenses^5^. Recent advancements in research focus on innovative BLDC motor speed control techniques that can address the above challenges. Despite their widespread use, traditional techniques such as proportional-integral-derivative (PID) control can suffer from non-linearities and dynamic load situations^6^. On the other hand, sophisticated methods like Fuzzy Logic Control (FLC), Sliding Mode Control (SMC), and Model Predictive Control (MPC) have demonstrated promise in improving motor performance under various operating conditions^7–13^.

In particular FLC has gained interest because of its resilience to non-linearities and uncertainties, which makes it a strong option for EV applications^14^. There are still a number of gaps in the literature despite these developments. For example, sensor-based control techniques are more expensive and complex, but they provide great precision^15^. On the other hand, sensorless control methods reduce the dependency on hardware, but they are vulnerable to changes in parameters and external disruptions^16^. Furthermore, there is also ongoing research into integrating regenerative braking systems with sophisticated control algorithms, especially in order to maximise energy recovery and improve overall system efficiency^17^.

Advanced control techniques including Model Predictive Control (MPC), Sliding Mode Control (SMC), and Direct Torque Control (DTC) pose a number of difficulties for EV applications despite their greater performance. For example, MPC necessitates the real-time resolution of intricate optimisation issues, which is computationally demanding and challenging to execute in systems with limited resources^18^. Similar to this, SMC is less appropriate for extremely dynamic situations since it requires accurate system modelling and may have chattering effects, despite its robustness^19^. Despite its ability to respond quickly to torque, DTC is sensitive to changes in parameters and frequently produces large torque ripple^13^. In contrast, fuzzy logic control eliminates the need for intricate mathematical models and offers a reliable and computationally economical solution for nonlinear systems^20^. In EV applications, where load and environmental conditions can change quickly, its capacity to manage uncertainty and adjust to changing operating circumstances makes it especially appropriate^21^. Fuzzy logic control was chosen for this study because of these benefits as well as its simplicity and convenience of use.

Key performance parameters for various motor types, such as induction motors, brushed DC motors, permanent magnet synchronous motors (PMSM), and BLDC motors, are compared in Table 1 to further emphasise the benefits of BLDC motors in EV applications. The comparison demonstrates how BLDC motors are a better option for contemporary electric vehicles due to their higher efficiency, torque density, and control flexibility^3,4,7^. For instance, BLDC motors provide improved torque density, which is essential for EV propulsion systems, and have higher efficiency (90–95%) than brushed DC motors (75–85%). Their applicability for dynamic EV applications is boosted by their compatibility with sophisticated control schemes including field-oriented control and sliding mode control^5,22^.

The following are some of the key contributions of our research:

- To address limitations of traditional PID controllers, such as performance degradation under dynamic loads, a fuzzy logic controller (FLC) was developed for robust closed-loop speed regulation of BLDC motors in electric vehicles.
- BLDC motor dynamics are thoroughly modelled using state-space approaches, offering a solid basis for FLC implementation.
- Comparison of the proposed FLC with conventional PID controllers, emphasising enhancements in speed control, energy economy, and load-variability adaptability .
- The effectiveness of the FLC in practical EV applications has been validated by hardware experiments and simulations .
- insights on how to combine fuzzy logic control with regenerative braking systems, providing a technique to improve overall system efficiency and energy recovery.

This paper is organized as follows: Sect. 2 presents the literature review, Section 3discusses the State space model of BLDC motor, Sect. 4 describes the closed loop operation of BLDC motor for EV applications, Sect. 5 illustrates the simulation results and discussion, Sect. 6 demonstrates the Experimental results and discussion, Sect. 7 demonstrates the handling Real-Time Computational Constraints in FLC for Embedded EV Motor Controllers, followed by the conclusion and references.

**Table 1:** Comparison of different characteristics required for electric vehicles for BLDC motor with different motors.

Sl.No	Measure of Performance	Induction motor	Brushed DC motor	Permanent Magnet Synchronous motor	BLDC motor	Ref
1	Power Capacity(kW)	From Moderate to High	Moderate	From Moderate to High	From Moderate to High	^3,4^
2	Convergence with Electric Vehicle Systems	Adequate	Adequate	Amplified	Amplified	^1,3^
3	Efficiency in percentage	85–92%	75–85%	92–98%	90–95%	^4,7^
4	Control Approach	Voltage Control for Direct Torque Control (DTC)	Voltage Regulation	Field Oriented Control	Field Oriented Control or Slide Mode Control	^5,22^
5	Density of Torque (Nm/kg)	Modest	Modest	extremely high	Enhanced	^3,7^
6	Loudness Index	Moderate	Moderate	Low	Low	^4,14^
7	Range of Speed (RPM)	From Moderate to High	Modest	High	High	^4,6^
8	Functional Sturdiness	Modest	Modest	Higher	Higher	^7,15^
9	Dimensions and weight	Comparatively larger in size	Comparatively larger in size	Light weighted and compact in size	Light weighted and compact in size	^4,19^
10	Flexibility in Changing Demands	Good	Good	Excellent	Excellent	^7,18^
11	Heat Transferrence	Sufficient	Modest	Highly efficient	Highly efficient	^4,19^
12	Pricing	A reasonable	Low to Moderate	Between Moderate and High	High	^3,4^
13	Complexity of Controller	Simpler	Simpler	Complicated	Complicated	^7,19^
14	Regenerative Braking	Limited	Limited	Increased capacity	Increased capacity	^16,20^
15	Sensor Type	Sensorless	Brush and commutator	Encoders or Resolver	sensors with a Hall effect or encoders	^6,15^

## Literature review

According to recent studies, electric vehicles (EVs) are crucial for sustainable transportation, and their performance can only be improved with efficient motor control techniques. The International Energy Agency’s Global EV Outlook 2023 highlights the need for enhanced control approaches to maximize energy efficiency and integration with renewable sources, while also outlining the EVs’ explosive expansion. Additionally, Richardson (2013) examines how EVs and the electric grid interact, emphasizing the need for advanced modeling techniques to make this integration easier.

Studies by Husain et al. (2021) and Mohammad & Jaber (2022) compare and examine several electric drive systems, with a concentration on brushless DC (BLDC) motors because of their controllability and efficiency benefits. These studies emphasize how crucial effective control techniques are to optimizing electric motor performance in automotive applications. Furthermore, Mounica & Obulesu (2022) investigate hybrid power management techniques, demonstrating how various energy sources can be integrated and how intelligent control techniques can maximize fuel efficiency. Advanced electric machines and EV control systems are examined critically by Liu et al. (2021), who uncover robustness and efficiency flaws in existing approaches. Sensorless control methods for BLDC motors are discussed by Gupta & Pandey (2012), whose work stress the necessity of reliable and effective control algorithms. All of the studies show that even though control strategies have been better understood and improved, there is still a great need for new methods, especially those that use fuzzy logic, which has shown to be more flexible and effective under a variety of circumstances (Ahmed et al., 2018; Anget et al., 2019).

In general, there is agreement in the literature regarding the significance of developing control techniques for BLDC motors in EV applications. Even though a lot of research has compared conventional and modern control techniques, further empirical verification and investigation of fuzzy logic control’s practicality are required to close current gaps and improve the performance of electric vehicles. The research highlights how significant the brushless DC (BLDC) motors are to improving the efficiency and performance of electric vehicles (EVs). The exceptional efficiency, reliability, and compact size of BLDC motors make them popular and add to the overall efficacy of EV propulsion systems.

Sensitivity to changes in load, performance nonlinearity, and the requirement for exact speed control, however, continue to be significant challenges. According to several researchers, conventional control techniques, such as proportional-integral-derivative (PID) controllers, frequently fail to sustain optimal performance in dynamic situations, resulting in problems like overshoot and instability. To overcome these limitations, Fuzzy Logic Control (FLC) is emerging as a favorable option and is increasingly gaining popularity. The flexibility and uncertainty-management capabilities of FLC make it an appealing substitute that enhances control accuracy and responsiveness in real-time applications. The operational efficiency and dependability of BLDC motors in electric vehicles might be greatly improved by FLC by successfully filling the gaps in current control approaches. This would open the door for more sophisticated and effective EV technologies. The outcomes from a number of research demonstrate that FLC is more versatile than typical PID control when it comes to managing non-linear circumstances. These studies demonstrate smoother speed transitions and less torque ripple. By offering a comparative performance study and confirming conclusions using simulation and experimental data, this work expands on these findings.

Table 2 provides an extensive summary of current approaches and highlights the latest advancements in this area of study.

**Table 2:** Summary of related researches for BLDC motor in EVs.

References	Control Method	Key Findings and challenges	Gap identified
Richardson (2013)^2^	Methods of Modeling	examines the effects of EVs on the grid and the incorporation of renewable energy	Advanced control techniques are required to address integration issues.
Husain et al. (2021)^3^	Technologies for Electric Drives	Challenges and trends in electric drives	Disparities in control robustness and efficiency among different drive types.
Mohammad & Jaber (2022)^4^	Comparative Evaluation of various electric motors for EVs	explains several EV electric motors and highlights the benefits of BLDC.	Comprehensive control strategies specifically designed for BLDC motors are required.
Mounica & Obulesu (2022)^5^	Power Management in Hybrid Energy systems for EVs	Techniques for combining several energy sources	Intelligent control methods are required to be addressed to to maximize energy use
Gupta & Pandey (2012)^6^	Sensorless BLDC motor control	examines the challenges associated with sensorless control methods.	Improved sensorless control techniques are required.
Liu et al. (2021)^7^	Evaluation of cutting-edge electrical machines for Evs	evaluates sophisticated machinery and control schemes	Specific case studies lacking
Baranowski et al. (2021)^14^	The evaluation of EMI	EMI mitigation for BLDC drives	development of reliable control schemes appropriate for practical situations.
Shokri & Naderi (2017)^15^	Affordable Drives	investigates basic BLDC motor drives.	Effective control algorithms that improve performance are required.
Nian et al. (2014)^16^	Regenerative Braking	explains EV regeneration systems.	Control challenges in implementing effective regenerative braking.
Neethu & Jisha (2012)^17^	Comparative Analysis	assesses different BLDC speed control techniques.	Evaluation of the efficacy of control methods in practical applications is required.
Ahmed et al. (2018)^23^	Comparing PI Control and Fuzzy Logic	Fuzzy logic improves speed control flexibility.	It is necessary to compare with a broader range of control strategies.
Anget et al. (2019)^24^	Fuzzy-Tuned PID	The efficiency of fuzzy tuning for speed stability is demonstrated.	requirement for fuzzy-tuned control systems to be tested in real-world scenarios.
Patel (2020)^25^	PID Tuning	explains how to tune PID control using the Ziegler-Nichols approach.	demand for sophisticated tuning methods tailored to various uses.
Wang et al. (2022)^8^	Dual Fuzzy Logic Systems	offers innovative fuzzy control techniques for BLDC motors.	investigation of resilience and flexibility under various operational circumstances.
Pamuji et al. (2021)^9^	Fuzzy Logic vs. ANN	compares BLDC motor control designs.	Control strategies must be empirically validated in a variety of settings.
Naqvi et al. (2024)^10^	Multi-objective PI Optimization	emphasizes energy-saving techniques for BLDC control.	A better understanding of multi-objective optimization techniques is needed.
Mopidevi et al. (2024)^11^	PI and ANFIS Comparison	A comparison of performance that suggests the possibility of hybrid approaches	It is necessary to thoroughly assess the effectiveness of hybrid control techniques.
Apribowo et al. (2021)^12^	Applications of Fuzzy Logic	evaluates fuzzy logic’s performance in BLDC motor control.	Fuzzy logic must be applied thoroughly in a variety of EV settings.
Sayed et al. (2024)^13^	Tilt Integral Derivative Control	presents novel control techniques to increase the BLDC motor’s efficiency.	New tactics must be validated and tested in real-world settings.
Potnuru et al. (2022)^20^	Salp Swarm Algorithm	Advanced optimization approaches for optimal speed control	Extensive research on algorithm performance under various circumstances is required.
Valle et al. (2020)^21^	Digital Control Techniques	explains methods for low inductance motor control.	Additional tactics are required for efficient control in applications with changing speed.

Different electric motors, each with unique benefits and drawbacks, have been investigated for EV applications. A comparison of various motor types according to significant performance metrics, as covered in earlier research, is given in Table 3.

**Table 3:** Comparison of electric motors for EV applications.

Sl.No	Motor Type	Advantages	Limitations	References
1	Induction Motor (IM)	Sturdy design and excellent dependability. The power capacity ranges from moderate to high. It is less expensive than PM motors. Appropriate for EV applications requiring high power.	Less efficient (85–92%) than PM motors. Complex control systems, such as Vector Control and DTC, are necessary. Increased generation of heat and energy losses.	^3,4,7^
2	Brushed DC Motor (BDC)	Easy to operate and control. High torque at startup. Easy to maintain and reasonably priced.	Reduced efficiency (75–85%). Brush wear and upkeep are high. A shorter lifespan and a reduced range of speeds.	^4,6,7^
3	Permanent Magnet Synchronous Motor (PMSM)	The highest efficiency, ranging from 92–98%. Compact design and high torque density. It runs smoothly and is precisely controlled. Ideal for EVs with strong performance.	High cost due to rare-earth magnets. Risk of demagnetization at high temperatures. Requires complex control strategies (FOC, SMC).	^3,7,19^
4	Brushless DC Motor (BLDC)	High efficiency (90–95%). Reduced maintenance (no brushes). Better heat dissipation due to its compact size. Enhanced torque-speed characteristics.	Expensive compared to brushed DC and induction motors. It needs precise electronic control. Sensor-based functionality (encoders, hall sensors).	^4,7,15^

From the Table 3 it is understood that for Electric Vehicle propulsion, BLDC motors are the best choice due to their attractive balance between efficiency, density of power, and control flexibility among the other motor types investigated. Even though PMSMs are slightly more efficient (92–98%), BLDC motors are still very efficient (90–95%) and have a number of benefits, such as a higher torque-to-weight ratio, less maintenance because they don’t require brushes, and accurate speed control through sophisticated control methods like Field-Oriented Control (FOC). Furthermore, BLDC motors provide regenerative braking, which enhances energy efficiency. They are a desirable choice for contemporary EV applications due to their small size, lightweight design, and affordability.

## Mathematical modelling of BLDC motor

State-space modeling provides a mathematical representation of the dynamic behavior of a Brushless DC (BLDC) motor, which is essential for improving control strategies in electric vehicle applications. The state-space model is crucial for creating efficient control schemes since it offers a comprehensive explanation of the motor’s behavior. In order to express the BLDC motor’s governing equations in phase variables, the following equation is obtained:

1 $$\:\left[\begin{array}{c}{V}_{RS}\\\:{V}_{YS}\\\:{V}_{BS}\end{array}\right]=\left[\begin{array}{ccc}{R}_{st}&\:0&\:0\\\:0&\:{R}_{st}&\:0\\\:0&\:0&\:{R}_{st}\end{array}\right]\left[\begin{array}{c}{i}_{rs}\\\:{i}_{ys}\\\:{i}_{bs}\end{array}\right]+\frac{d}{dt}\left[\begin{array}{ccc}{L}_{rr}&\:{L}_{ry}&\:{L}_{rb}\\\:{L}_{yb}&\:{L}_{yy}&\:{L}_{yb}\\\:{L}_{br}&\:{L}_{by}&\:{L}_{bb}\end{array}\right]\left[\begin{array}{c}{i}_{rs}\\\:{i}_{ys}\\\:{i}_{bs}\end{array}\right]+\left[\begin{array}{c}{e}_{r}\\\:{e}_{y}\\\:{e}_{b}\end{array}\right]$$

Where $\:{V}_{RS},{V}_{YS},{V}_{BS}$ are the phase voltages of the stator windings, $\:{R}_{st}$ is the resistance of the stator,$\:{i}_{rs},{i}_{ys},{i}_{bs}$ are the phase currents of the stator windings, $\:{L}_{rr},{L}_{yy},{L}_{bb}$ are the self-mutual inductances of the phases R, Y and B respectively.$\:{L}_{ry},{L}_{yb}and\:{L}_{br}$ are the mutual inductances among the phases R, Y and B respectively. Similarly, $\:{e}_{r},{e}_{y}\:and\:{e}_{b}$ are the phase values of the back EMFs. It has been presumed that each and every winding has the similar resistance. Furthermore, it has been presumed that the rotor reluctance is expressed as follows if there isn’t an apparent rotor and it stays constant along an angle,

2 $$\:{L}_{rr}={L}_{yy}={L}_{bb}={L}_{st}$$

and similarly,

3 $$\:{L}_{ry}={L}_{yb}={L}_{br}={L}_{yr}={L}_{by}={L}_{rb}={M}_{st}$$

Substituting Eqs. (2) and (3) in Eq. (1), we get,

4 $$\:\left[\begin{array}{c}{V}_{RS}\\\:{V}_{YS}\\\:{V}_{BS}\end{array}\right]=\left[\begin{array}{ccc}{R}_{st}&\:0&\:0\\\:0&\:{R}_{st}&\:0\\\:0&\:0&\:{R}_{st}\end{array}\right]\left[\begin{array}{c}{i}_{rs}\\\:{i}_{ys}\\\:{i}_{bs}\end{array}\right]+\frac{d}{dt}\left[\begin{array}{ccc}{L}_{st}&\:{M}_{st}&\:{M}_{st}\\\:{M}_{st}&\:{L}_{st}&\:{M}_{st}\\\:{M}_{st}&\:{M}_{st}&\:{L}_{st}\end{array}\right]\left[\begin{array}{c}{i}_{rs}\\\:{i}_{ys}\\\:{i}_{bs}\end{array}\right]+\left[\begin{array}{c}{e}_{r}\\\:{e}_{y}\\\:{e}_{b}\end{array}\right]$$

Since the phase currents of the stator windings are balanced, i.e. $\:{i}_{rs}+{i}_{ys}+{i}_{bs}=0$, then we can simplify the inductances as follows,

5 $$\:{M}_{st}{i}_{ys}+{M}_{st}{i}_{bs}=-{M}_{st}{i}_{rs}$$

The electromagnetic torque that is developed is given by,

6 $$\:{T}_{em}=\frac{\left({e}_{r}{i}_{rs}+{e}_{y}{i}_{ys}+{e}_{b}{i}_{bs}\right)}{{\omega\:}_{me}}$$

The back ems are trapezoidal in nature and are described by the following equation,

7 $$\:\left[\begin{array}{c}{e}_{r}\\\:{e}_{y}\\\:{e}_{b}\end{array}\right]={\omega\:}_{me}{\lambda\:}_{me}\left[\begin{array}{c}{f}_{rs}\left({\theta\:}_{ro}\right)\\\:{f}_{ys}\left({\theta\:}_{ro}\right)\\\:{f}_{bs}\left({\theta\:}_{ro}\right)\end{array}\right]$$

Where $\:{\omega\:}_{me}\:is\:$the angular speed in radians per seconds is, $\:{\lambda\:}_{me}$ is the flux linkages, $\:{\theta\:}_{ro}$ is the rotor position in radians. The functions $\:{f}_{rs},{f}_{ys},\:{f}_{bs}$ have the same shapes as that of the back ems.

The dynamic equation of the motor-load system with intertia $\:{J}_{b}$, friction coefficient, $\:{B}_{b}$ and the load torque, $\:{T}_{L\:\:}$is given by,

8 $$\:{J}_{b}\frac{d{\omega\:}_{me}}{dt}+{B}_{b}{\omega\:}_{me}={T}_{em}-{T}_{L}$$

Position and speed of the electrical rotor are inter related by,

9 $$\:\frac{d{\theta\:}_{ro}}{dt}=\frac{P}{2}{\omega\:}_{me}$$

where P is the motor pole number.

When all pertinent equations are combined, the system’s state-space model is given by,

10 $$\:\dot{x}\left(t\right)=Ax\left(t\right)+Bu\left(t\right)+Ce\left(t\right)$$

Where the state variables are given by,

11 $$\:x\left(t\right)=\left[\begin{array}{ccc}{i}_{rs}&\:{i}_{ys}&\:\begin{array}{ccc}{i}_{bs}&\:{\omega\:}_{me}&\:{\theta\:}_{ro}\end{array}\end{array}\right]$$

Consequently, the system matrices shown below,

12 $$\:A=\left[\begin{array}{ccccc}\frac{{-R}_{st}}{{L}_{st}-{M}_{st}}&\:0&\:0&\:-\frac{{\lambda\:}_{me}}{{J}_{b}}{f}_{rs}\left({\theta\:}_{ro}\right)&\:0\\\:0&\:\frac{{-R}_{st}}{{L}_{st}-{M}_{st}}&\:0&\:-\frac{{\lambda\:}_{me}}{{J}_{b}}{f}_{ys}\left({\theta\:}_{ro}\right)&\:0\\\:0&\:0&\:\frac{{-R}_{st}}{{L}_{st}-{M}_{st}}&\:-\frac{{\lambda\:}_{me}}{{J}_{b}}{f}_{bs}\left({\theta\:}_{ro}\right)&\:0\\\:\frac{{\lambda\:}_{me}}{{J}_{b}}{f}_{rs}\left({\theta\:}_{ro}\right)&\:\frac{{\lambda\:}_{me}}{{J}_{b}}{f}_{ys}\left({\theta\:}_{ro}\right)&\:\frac{{\lambda\:}_{me}}{{J}_{b}}{f}_{bs}\left({\theta\:}_{ro}\right)&\:\frac{-{B}_{b}}{{J}_{b}}&\:0\\\:0&\:0&\:0&\:\frac{P}{2}&\:0\end{array}\:\:\right]$$

13 $$\:B=\left[\begin{array}{cccc}\frac{1}{{L}_{st}-{M}_{st}}&\:0&\:0&\:0\\\:0&\:\frac{1}{{L}_{st}-{M}_{st}}&\:0&\:0\\\:0&\:0&\:\frac{1}{{L}_{st}-{M}_{st}}&\:0\\\:0&\:0&\:0&\:\frac{1}{{L}_{st}-{M}_{st}}\end{array}\right]$$

14 $$\:C=\left[\begin{array}{ccc}\frac{1}{{L}_{st}-{M}_{st}}&\:0&\:0\\\:0&\:\frac{1}{{L}_{st}-{M}_{st}}&\:0\\\:0&\:0&\:\frac{1}{{L}_{st}-{M}_{st}}\end{array}\right]$$

15 $$\:u=\left[\begin{array}{c}{V}_{RS}\\\:{V}_{YS}\\\:{V}_{BS}\\\:{T}_{L}\end{array}\right]$$

16 $$\:e=\left[\begin{array}{c}{e}_{r}\\\:{e}_{y}\\\:{e}_{b}\end{array}\right]$$

Thus the above equations describe the state modeling of a BLDC motor^26^.

The Fuzzy Logic Controller’s (FLC) performance is largely dependent on the accuracy of the state-space model. Its impact is demonstrated by the following reasons:

- The FLC can make better control decisions based on real system behaviour when the state-space model is more accurate because it gives a clear mathematical picture of the system dynamics.
- FLC makes control decisions based on fuzzy rules. Suboptimal control actions may result from input variables (error and its derivative) that do not adequately reflect actual system conditions if the model is not representative of the system states.
- A precise model guarantees that the FLC parameters are adjusted efficiently, improving stability and resilience. Inaccurate control actions brought on by an inadequate model could lead to oscillations or decreased performance.
- The FLC can react to fluctuations and changes in system parameters more effectively and precisely when it has a well-defined state-space model.
- The simulated performance of the FLC will be more in line with experimental findings if the state-space model closely resembles the behaviour of real-world systems. This will lessen the need for intensive real-world testing and fine-tuning.

## Closed loop operation of BLDC motor for EV applications

In order to develop efficient closed-loop control systems in electric vehicles (EVs), it is essential to understand the dynamic behavior of BLDC motors as determined by mathematical modeling. Optimizing control techniques and guaranteeing accurate torque, position, and speed management are made possible by having a solid understanding of the state-space representation and equations regulating the motor’s operation.

The closed-loop control system block diagram for a BLDC motor in an electric vehicle is shown in Fig. 3. In an electric vehicle (EV), a closed-loop control system for a Brushless DC (BLDC) motor uses feedback techniques to optimize and manage the motor’s performance. The objective is to maintain exact control over the motor’s torque, position, and speed to ensure the electric vehicle runs smoothly and effectively. The following is an explanation of the closed-loop operation:

Different kinds of sensors are used to collect vital data regarding the functioning of the motor. Typical sensors include a position sensor (such as a Hall effect sensor) to ascertain the rotor position, a speed sensor to measure the motor’s speed, and occasionally a temperature sensor to track the motor’s temperature. The data from the sensors is processed by a digital signal processor (DSP) or microcontroller in the control system. This controller functions as the brain of the system, making decisions depending on input. The system requires a reference input, which is frequently set by the driver or the control system of a vehicle. This could be the required speed, position, or torque. The controller compares the actual motor state as determined by the sensors with the intended reference input. This information is used to calculate the signal of error, which shows the discrepancy between the desired and actual conditions.

The controller processes the error signal and decides how to modify the motor action to minimise the error using a control method, generally known as a proportional-integral-derivative, or PID, controller. The power electronics of the motor receive control signals from the controller, which modify the electrical current passing through the motor windings. Consequently, the motor’s speed, position, or torque are adjusted to align it with the desired reference input. The sensors are always giving the controller feedback, hence the process is continuous.

The controller makes adjustments in real time to maintain the motor’s intended level of operation. By making sure the BLDC motor operates efficiently and dynamically adjusts to variations in load or operating conditions, this closed-loop control system contributes to assuring the overall performance and stability of the electric vehicle.

**Fig. 1:**
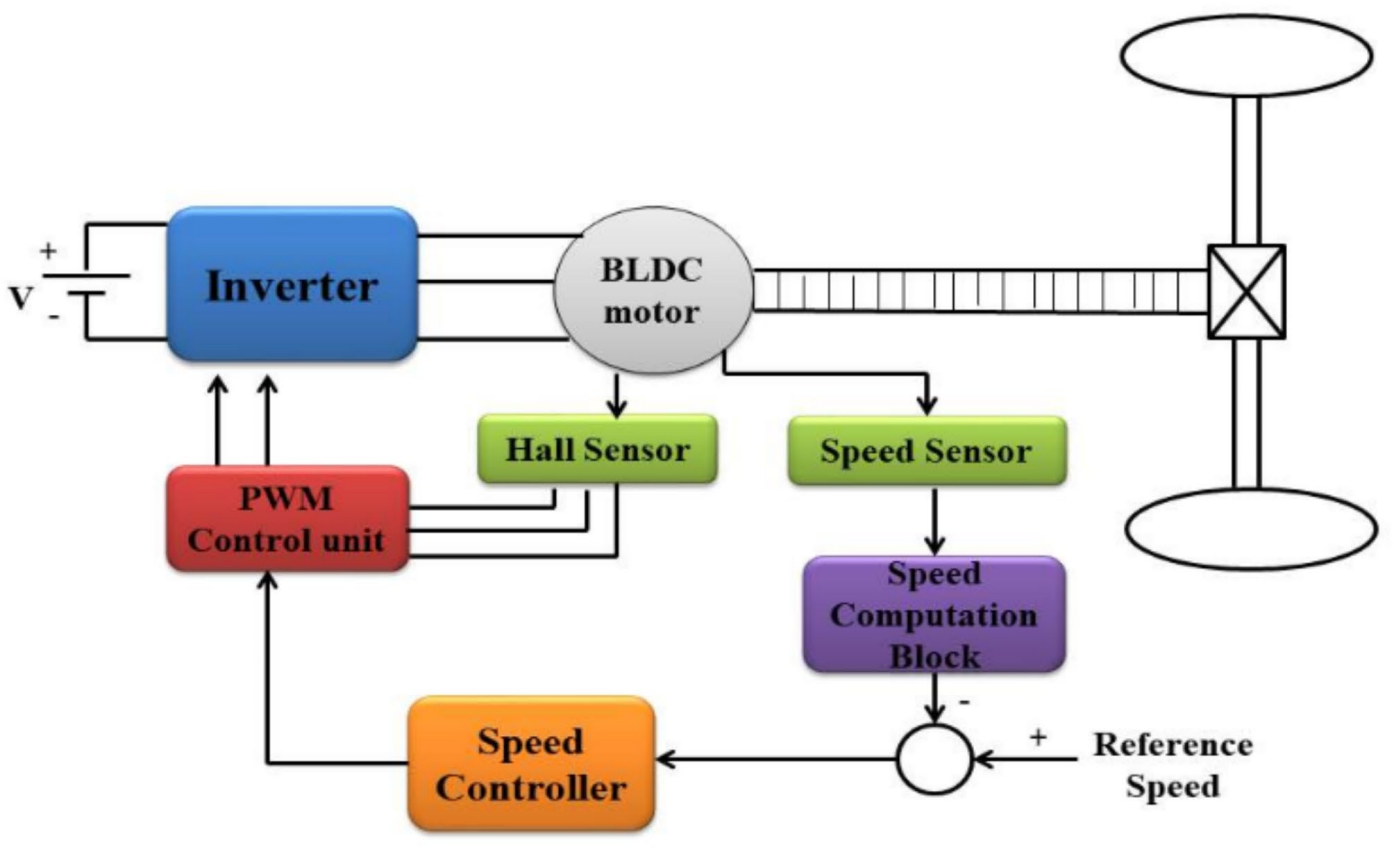
Closed-Loop control of Electric vehicle drive system with BLDC motor.

The operational workflow and complete control interface of the suggested speed control system for the Brushless DC (BLDC) motor in electric vehicle applications are shown in Fig. 1. The components, their interconnections, and pertinent mathematical models are covered in detail in the sections that follow.

### Information flow and component interaction

#### Inverter stage

To power the BLDC motor, the inverter transforms the DC supply into the three-phase AC output that is needed. Pulse Width Modulation (PWM) signals produced by the control algorithm control this stage. To create the necessary phase currents, the inverter alternates the current flowing across the motor windings using semiconductor switches, usually IGBTs or MOSFETs. The inverter’s switching function can be represented mathematically as follows:

17 $$\:{V}_{rn}={S}_{r}{V}_{dc},\:{V}_{yn}={S}_{y}{V}_{dc},\:{V}_{bn}={S}_{b}{V}_{dc}$$

Where $\:{S}_{r}\:,\:{S}_{y}\:,\:{S}_{b}$ indicate the DC link voltage and the switching states (0 or 1) for each phase.

#### BLDC motor dynamics

The inverter generates phase currents that power the BLDC motor. The phase-specific dynamic voltage equation is as follows:

18 $$\:{V}_{a}={R}_{a}{I}_{a}+{L}_{a}\frac{d{I}_{a}}{dt}+{E}_{a}$$

where Va is the applied phase voltage, Ra is the resistance per phase, La is the inductance per phase, Ia is the phase current and Ea is the back emf. The back emf is given by,

19 $$\:{E}_{a}={K}_{e}{\omega\:}_{m}$$

where Ke is the back emf constant and $\:{\omega\:}_{m}$ is the angular velocity of the rotor.Hall and Speed Sensors.

#### Hall and speed sensors

For commutation, the rotor position feedback provided by the Hall sensors is crucial. By measuring the time between successive Hall sensor signals, the speed sensor determines the motor speed. The speed control block receives the feedback signals, which are continuously monitored, and makes dynamic modifications.

### Control algorithm interface

#### Speed controller

The speed error signal, which is calculated as the difference between the reference speed and the observed speed, is sent to the speed controller:

20 $$\:e\left(t\right)=\:{\omega\:}_{ref}-{\omega\:}_{m}$$

The following control law is used by a proportional-integral-derivative (PID) controller to generate the control input:

21 $$\:u\left(t\right)=\:{K}_{p}e\left(t\right)+{K}_{i}\underset{0}{\overset{t}{\int\:}}e\left(\tau\:\right)d\tau\:+{K}_{d}\frac{de\left(t\right)}{dt}$$

Where Kp is the proportional gain, Ki is the integral gain and Kd is the derivative gain. u(t) is the control input that establishes the PWM signals’ duty cycle that power the inverter.

#### PWM control unit

The PWM control unit converts the inverter switches’ duty cycles from the control input. This procedure creates the necessary PWM signals by comparing the control signal with a high-frequency carrier waveform.

### Mathematical model for the BLDC motor

The motor’s electromagnetic torque is proportional to the phase current and is given by,

22 $$\:{T}_{e}=\:{K}_{T}{I}_{a}$$

and the governing equations of the motor load dynamics is given by,

23 $$\:{T}_{e}-{T}_{L}=J\frac{d{\omega\:}_{m}}{dt}+B{\omega\:}_{m}$$

Where K_T_ is the torque constant, T_L_ is the load torque, J is the moment of inertia and B is the damping ratio.

### Feedback loop

The control system maintains the desired motor speed by operating in a closed-loop arrangement. The following are the feedback loop’s main phases:

- Speed Error Calculation: To calculate the speed error, the measured speed and the reference speed are compared.
- Control Action: To provide the control input, the PID controller analyses the speed error.
- PWM Generation: To power the inverter, the control input is transformed into PWM signals.
- Motor Response: To run the motor at the required speed, the inverter output regulates the phase currents.
- Monitoring: To provide precise and dynamic speed management, the rotor position and speed are continuously monitored.

By constantly adapting to changes in load, supply voltage, and other disturbances, this closed-loop technology guarantees reliable speed control.

Let’s discuss PID controller and Fuzzy logic controller based speed control operation in detail^22^.

#### PID controller

PID controllers efficiently regulate motor speed by using proportional, integral, and derivative components in feedback loops. PID controllers have been shown to be capable of managing dynamic fluctuations, which guarantees BLDC motors have reliable and responsive speed control. Their flexibility to adjust to various operating circumstances makes them a preferred choice in many industrial applications.Optimization of PID parameters are crucial which enhances system responsiveness and performance, making them critical in electric vehicles. These parameters in turn maintains consistent speed control. Additionally, external factors like temperature and load variations might impact performance, necessitating regular modifications and fine-tuning.

The output of a PID controller can be mathematically expressed as,

24 $$\:u\left(t\right)=\:{k}_{p}e\left(t\right)+{k}_{i}{\int\:}_{0}^{t}e\left(\tau\:\right)d\tau\:+{k}_{d}\frac{de\left(t\right)}{dt}$$

where $\:{k}_{p}$ is the proportional gain, $\:{k}_{i}$ is the integral gain, and $\:{k}_{d}$ is the derivative gain. To achieve the desired system performance, these parameters must be properly tuned^25^. There are several ways to get the ideal values for $\:{k}_{p}$, $\:{k}_{i}$ and $\:{k}_{d}$, including model-based techniques and trial-and-error. Table 4 summarizes the Ziegler-Nichols method’s tuning parameters based on the plant’s transient response characteristics.

**Table 4:** ZN tuning rules.

Controllers	Kp	T_i_	T_d_
P-type	$\:\frac{{T}_{z}}{{L}_{z}}$	$\:\infty\:$	0
PI-type	$\:0.9\frac{{T}_{z}}{{L}_{z}}$	$\:\frac{{L}_{z}}{0.3}$	0
PID-type	$\:1.2\frac{{T}_{z}}{{L}_{z}}$	$\:{2L}_{z}$	$\:{0.5L}_{z}$

#### Fuzzy logic controller

**Fig. 2:**
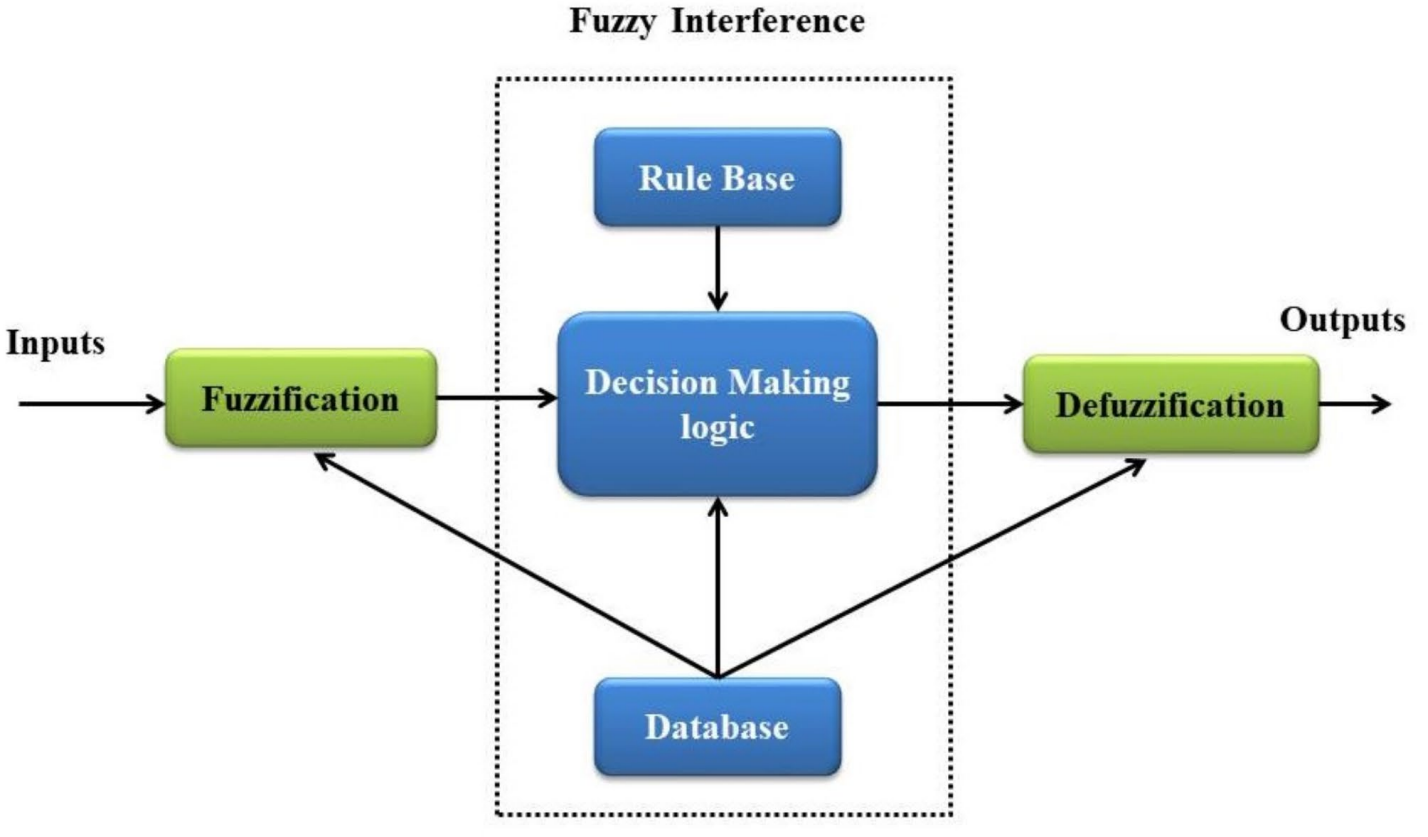
Fuzzy Logic Controller.

Performance can be improved when BLDC motors are integrated with a fuzzy logic controller (FLC), particularly in non-linear and unexpected systems. FLCs use a rule-based methodology that converts input variables into control signals by utilizing expert knowledge to efficiently handle variations in load and voltage. This technique minimizes sudden changes in motor operation, which is essential for applications requiring high precision, and enables FLCs to deliver smooth and accurate control. Because of their adaptability, they can be adjusted to maximize motor performance under varying operating circumstances. Furthermore, FLCs can be integrated with other control techniques to produce hybrid systems that enhance stability and response times substantially. Figure 2 depicts the fuzzy logic controller’s schematic diagram.

The FLC functions in three main stages:

1. Fuzzification: Transforms clear input data into fuzzy sets, expressing the imprecision and ambiguity of the real world. The FLC is more adaptable than conventional controllers since it can represent a greater range of input situations by giving membership degrees to input variables.
2. Fuzzy Inference: interprets fuzzy input using pre-established rules, allowing for context-aware decision-making. This procedure improves overall performance by enabling the controller to make informed decisions based on past data and present conditions.
3. Defuzzification: Converts fuzzy output into accurate control signals by applying weighted average or centroid methods. This stage guarantees that the output is both comprehensible and actionable, allowing the FLC to successfully use control strategies in practical situations.

In applications where precise mathematical modeling is difficult, FLCs are extremely beneficial in improving the stability and effectiveness of BLDC motor control systems. They are an essential technology in contemporary motor control applications because of their capacity to effectively control under a variety of circumstances and handle uncertainty.

## Simulation results and discussion

MATLAB/Simulink is used to simulate BLDC motor speed control utilising both fuzzy logic controller and traditional PID controller The BLDC motor used for analysis is of 2 kW capacity with voltage rating of 48 V and the rated speed of 1500 rpm which is considered for the EV application. The following presumptions guide the design of the fuzzy logic controller scheme:

The error (e) and change in error (∆e) are the input variables taken into consideration. The difference between the BLDC motor’s actual speed and the intended (reference) speed is known as the error, and the error signal’s rate of change is known as the error signal change. Similarly the output variables are considered as the change in duty cycle$\:(\varDelta\:Duty)$ and it is defined as the rate of variation in the Pulse Width Modulation (PWM) signal’s duty cycle that is sent to the motor controller. The membership function and the rule base for the BLDC motor speed control using FLC is given in Table 5.

**Table 5:** Fuzzy logic control rule base.

$\:\text{e}/\varDelta\:\text{e}$	NB	NM	NS	ZO	PS	PM	PB
NB	PB	PB	PM	PM	PM	ZO	ZO
NM	PB	PM	PM	PM	ZO	ZO	ZO
NS	PM	PM	PM	ZO	ZO	ZO	NM
ZO	PM	PM	ZO	ZO	ZO	NM	NM
PS	PM	ZO	ZO	ZO	NM	NM	NM
PM	ZO	ZO	ZO	NM	NM	NM	NB
PB	ZO	ZO	NM	NM	NM	NB	NB

Where NB is Negative Big, NM is Negative Medium, NS is Negative Small, ZO is Zero, PS is Positive Small, PM is Positive Medium, and PB is Positive Big.

**Fig. 3:**
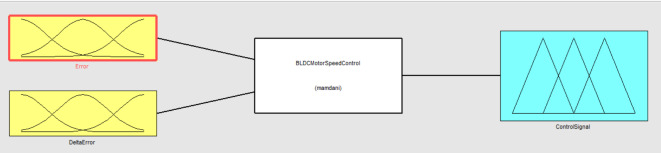
Block diagram of Fuzzy Logic Controller.

**Fig. 4:**
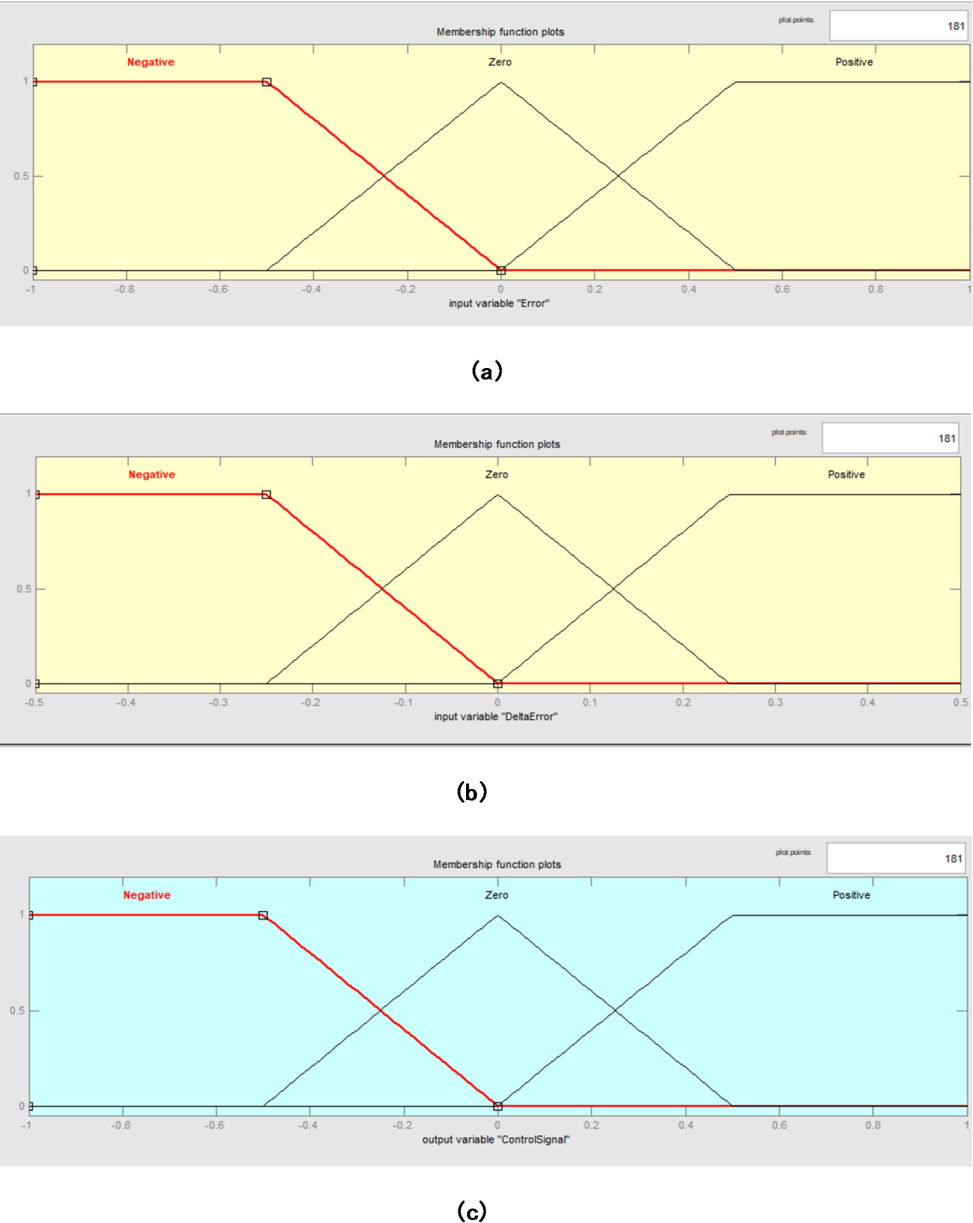
(**a**). Membership functions for the Input Variable “Error”. (**b**) Membership functions for the Input Variable “DeltaError”. (**c**) Membership functions for the Output Variable “ControlSignal”.

From Table 4 it is understood that the each input and output variable is given a linguistic name (e.g., NB, NM, NS, ZO, PS, PM, PB) to represent a particular state or situation. Fuzzifying the inputs and outputs for fuzzy logic processing is aided by membership functions, which specify the extent to which a value belongs to each linguistic word. The rule base establishes the mapping between linguistic terms for the input variables and their combinations for the output variable. The linguistic term for the output variable is contained in each cell of the rule base table, and it is determined by combining the linguistic terms for the input variables. A common algorithm that specifies the optimal crisp value based on a fuzzy set is the centre of area or centroid approach, which is used to find the area of fuzzy sets in order to achieve the crisp output. The block diagram of the fuzyy logic controller for BLDC speed control implemented using MATLAB/Simulink is shown in Fig. 3. From Fig. 3 it is understood that the error and change in error are considered as input variables and the duty cycle is considered as the output variable. Based on the membership function as illustrated in Table 4, the rules are defined and are executed. The membership functions for the input, error input and the control output are as illustrated in Fig. 4 (a),4(b) and 4(c) respectively.

To ascertain the motor’s true speed at any given moment, real-time feedback on the rotor position and speed is provided by the Hall sensor data. Following a comparison of this speed data with the reference or intended speed, the Fuzzy Logic Controller (FLC) receives two crucial inputs:

(i) Error (): The discrepancy between the actual speed determined by the Hall sensors and the reference (intended) speed.
(ii) Error Change (Δ): The rate at which the error is changing in relation to the target speed, indicating whether the motor is speeding up or slowing down.

Then, using the predefined membership functions displayed in Fig. 7(a) and 7(b), these inputs, and Δe, are fuzzified (transformed into fuzzy linguistic variables). Linguistic phrases that characterize the system’s state, such as Positive Small (PS), Zero (ZO), or Negative Big (NB), are linked to each input.

## Utilizing the fuzzy rule foundation

Following fuzzification, the inputs are compared to the rule base (Table 4), where each rule associates a particular combination of e and Δe with an output, in this case the duty cycle change (ΔDuty). In essence, this set of rules specifies how the controller should react to every possible combination of speed error and error change rate in order to get the motor speed closer to the target value. For instance, the rule base may produce a Positive Big (PB) for ΔDuty, suggesting a big increase in the PWM duty cycle to swiftly boost motor speed, if the speed is much below the reference speed (huge negative error) and the error is increasing (positive Δe).

## PWM control signal generation

The fuzzy output is then transformed into a clear, actionable value by defuzzifying the output variable, ΔDuty. The PWM duty cycle, which regulates the average voltage and power delivered to the motor, can be precisely adjusted by the defuzzified output. The PWM signal that powers the motor is directly derived from this value, which was obtained from the rule base and improved through defuzzification. The output membership functions specify how much change can be made to the PWM duty cycle, as seen in Fig. 7(c). The PWM signal then applies this modification to the motor driver circuit, which determines the motor’s power level and speed.

## Periodic feedback loop

By continuously receiving position and speed data from the Hall sensors, the FLC modifies the duty cycle in response to current conditions, and the motor reacts to these modifications, creating a continuous feedback loop. The technology provides dynamic and responsive control by repeating this procedure if the motor speed deviates once more. In conclusion, the measurement of actual speed and the creation of error signals depend on the data from Hall sensors. In order to maintain exact speed control, the FLC rule base uses these signals to calculate the ideal duty cycle adjustment, which is then translated to the PWM output that powers the motor.

**Fig. 5:**
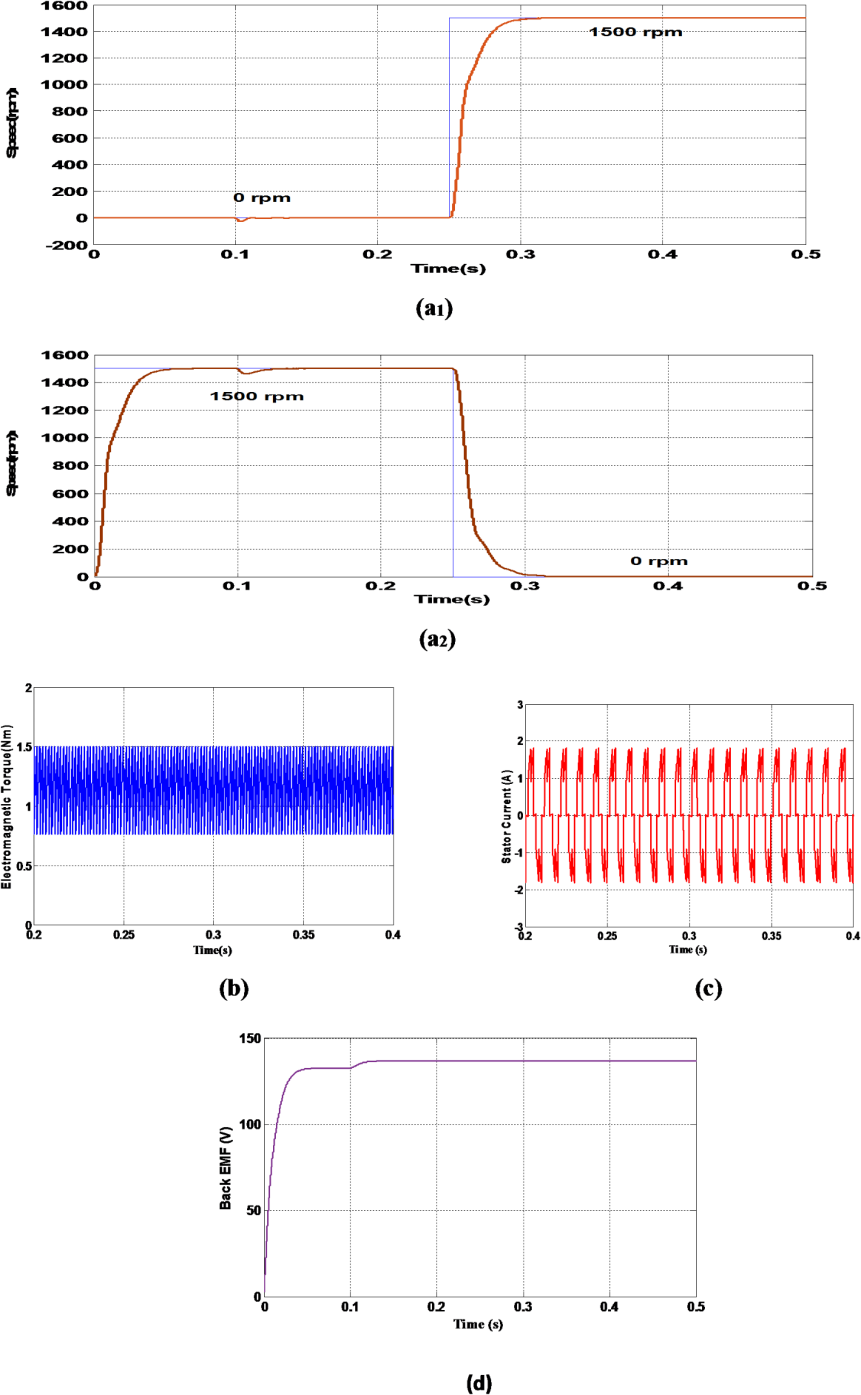
(**a**_**1**_,**a**_**2**_,**b**,**c**) Rotor speed, Electromagnetic torque and Stator current of BLDC motor with Fuzzy Logic controller. (**d**) Back EMF (FLC).

The results thus obtained for the above defined rules such as speed, torque, stator current and stator back emf are shown in the Fig. 5 (a), 5(b), 5(c) & 5(d) respectively. The speed of the motor is varied for two different values such as 0 rpm and 1500 rpm and also from 1500 rpm to 0 rpm as shown in Fig. 5 (a_1_) and Fig. 5(a_2_) respectively. The BLDC motor along with Fuzzy logic controller is able to achieve the desired speed at a very lesser time interval with a settling time of 0.05 s with very less or nearly zero steady state error. The speed waveform shows no overshoot or undershoots and the speed control thus obtained is dynamic and smooth, which is more desirable for the EV applications. The transition from one range of speed to other i.e. from 1500 rpm to 0 rpm and vice versa is taking place instantaneously and in a smoother fashion which is an added benefit for the EVs.

Figure 5(b) shows the Electromagnetic torque waveform which shows a stable and steady variation without any fluctuations. The stator currents and the back emf are illustrated in the Fig. 5(c) and Fig. 5 (d) respectively.

**Fig. 6:**
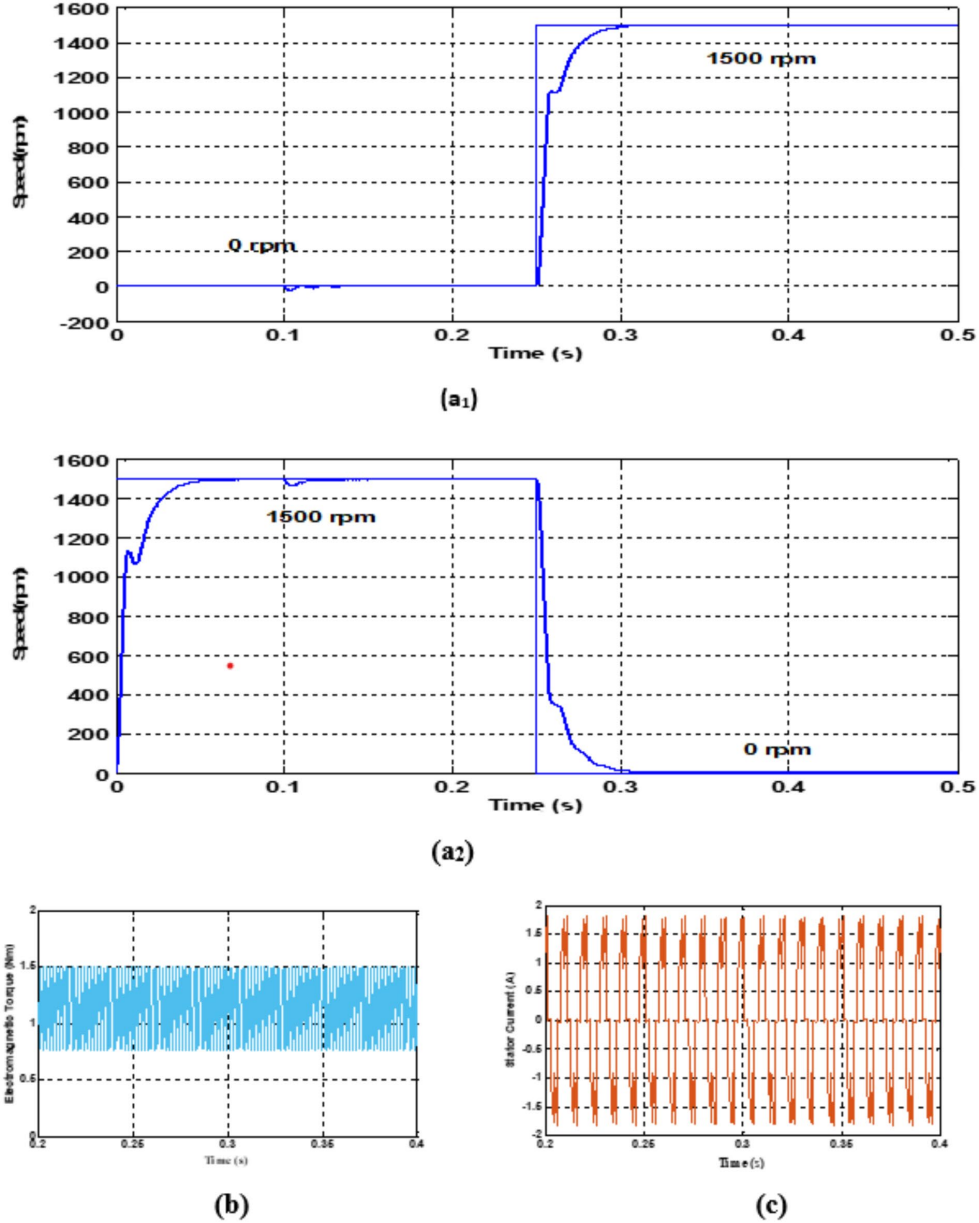

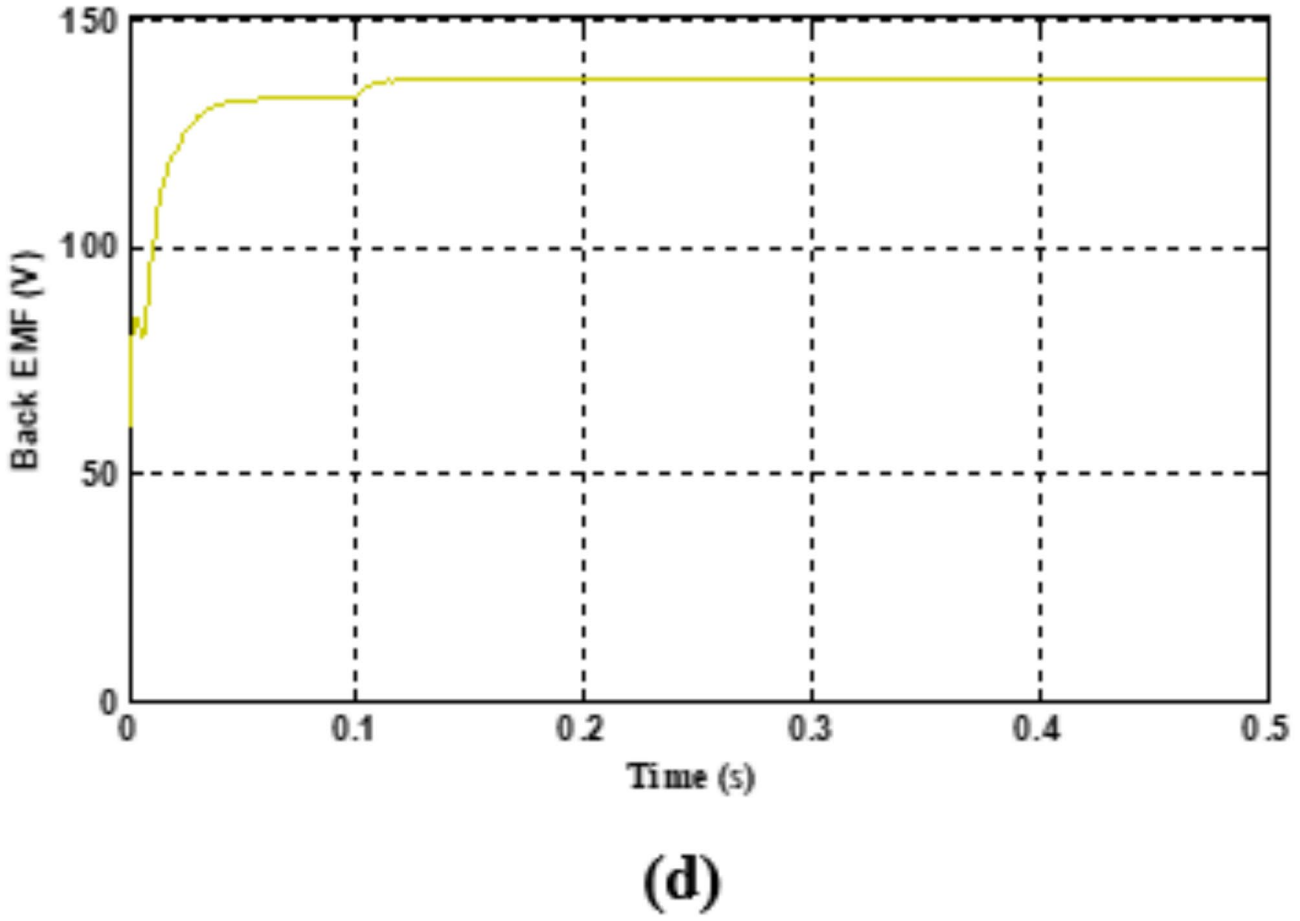
(**a**,**b**,**c**) Rotor speed, Electromagnetic torque and Stator current of BLDC motor with PID controller. (**d**) Back EMF (PID Controller).

The corresponding speed, torque, stator currents and back emf waveforms for the BLDC motor with conventional PID controller is shown in Fig. 6(a),6(b),6(c) & 6(d) respectively.

Figure 6a1 illustrates the speed response during start up of the BLDC motor obtained using conventional PID controller, When compared against that obtained using FLC (shown in Fig. 5a1), it is found that, FLC enables the motor to reach 1500 rpm with a faster rise time and minimal overshoot. FLC achieves the steady-state speed in about 0.28 s, which is faster than that achieved usibg PID control. On ther other hand by investigating the speed reversal response shown in Fig. 6a2 obtained using PID control demonstrates that the the FLC-controlled motor decelerates faster, reaching zero speed in 0.32 s. Furthermore, FLC exhibits reduced undershoot during the transition and smoother dynamic performance.

The PID controller values thus designed are $\:{k}_{p}=0.02,\:{k}_{i}=10\:and\:{k}_{d}=2.22\times\:{10}^{-6}$ and the corresponding values of Zero Time constant ($\:{T}_{z}$) and the Lead Time constant ($\:{L}_{z})$ are 0.000111 and 0.002 respectively. As is typical in complex or nonlinear systems like electric vehicles, the constants Tz and Lz derived from these gains are fixed for all operating situations, which could lead to less-than-ideal performance when the system dynamics change.

When compared with the conventional PID controller the fuzzy logic controller performs well. From Fig. 9, though performance of PID controller is quite appreciable in terms of tracking the desired speeds, it shows some evidences of overshoots and undershoots. The torque and current waveforms shows some fluctuations also. The time domain specifications of both the controllers are illustrated in Table 5. From Table 6, it is understood that the settling time of the speed response for the motor with FLC is very less of the order of 0.05s as against the PID controller, whose settling time is 0.1s. Further it is observed that there are no overshoots or undershoots and the steady state error is also nil. On the other hand if we observe the speed response of the PID controller, there occurs the steady state error of the order of 0.6 rpm to 1 rpm. Both the FLC and PID controller are capable of tracking the references appropriately and additionally there is a smooth transition between the speed changes were observed with FLC which is much desirable for Electric vehicle operation.

**Table 6:** Comparison of time domain specifications of BLDC motor with FLC and PID controller.

Ref. Speed	Actual Speed	PID Controller				Actual Speed	Fuzzy Logic Controller			
		Rise Time	Settling Time	Peak Over shoot	Steady State Error		Rise Time	Settling Time	Peak Over shoot	Steady State Error
1000	999.4	0.5	0.1	0	0.6	1000	0.01	0.05	0	0
1200	1199	0.55	0.15	0	1	1200	0.015	0.055	0	0
1500	1499	0.56	0.16	0	1	1500	0.015	0.06	0	0
2000	2000	0.7	0.16	0	0	2000	0.016	0.05	0	0
2500	2500	0.9	0.2	0	0	2500	0.02	0.05	0	0

In addition to the aforementioned, a thorough analysis of the fuzzy logic controller (FLC) has been carried out by comparing it with other control systems such as sliding mode control (SMC), and model predictive control (MPC). In terms of faster rise and settling durations, zero overshoot, and no steady-state error, FLC performs better while retaining a moderate level of computing complexity, as shown in Table 7. On the other hand, MPC and SMC demonstrate competitive dynamic responses despite having larger computing demands. These comparisons highlight FLC’s efficacy and efficiency in enhancing system control dynamics. Table 7 lists the main performance metrics and computational effectiveness for every control strategy along with pertinent research references.

**Table 7:** Comparative analysis of control strategies for system performance.

Control Strategy	Rise Time (s)	Settling Time (s)	Overshoot (%)	Steady-State Error	Computational Complexity	Key References
PID	0.5	0.1	5%	0.6 RPM	Low	[16], [22]
Fuzzy Logic (FLC)	0.01	0.05	0%	0 RPM	Moderate (Optimized)	This Study, [12]
Model Predictive Control (MPC)	0.02	0.06	2%	0.2 RPM	High	[18]
Sliding Mode Control (SMC)	0.03	0.07	1%	0.1 RPM	Moderate	[20]

## Experimental results and discussion

**Fig. 7:**
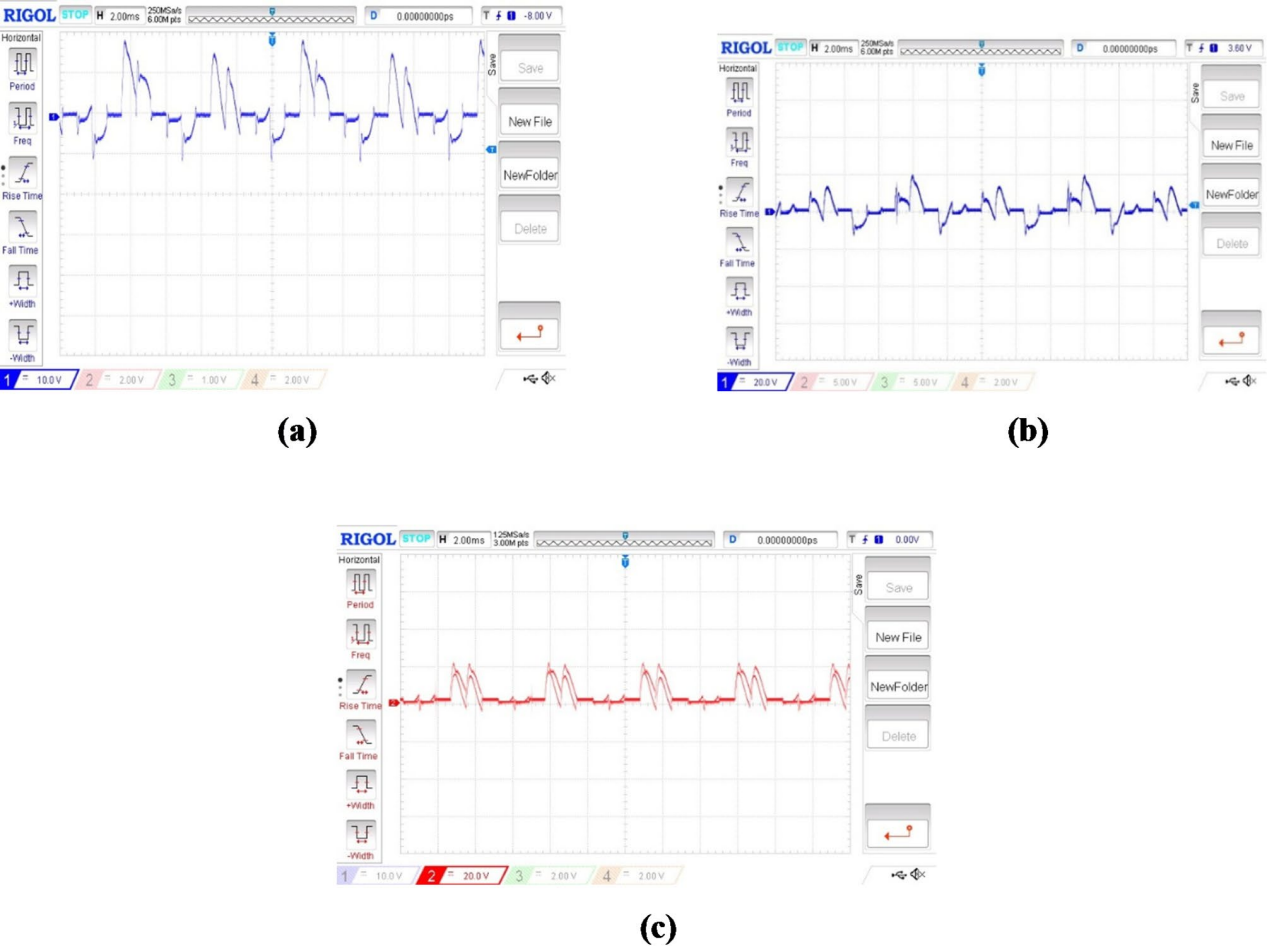
(**a**) Stator Current for phase R, (**b**) Stator Current for phase Y, (**c**) Stator Current for phase B.

**Fig. 8:**
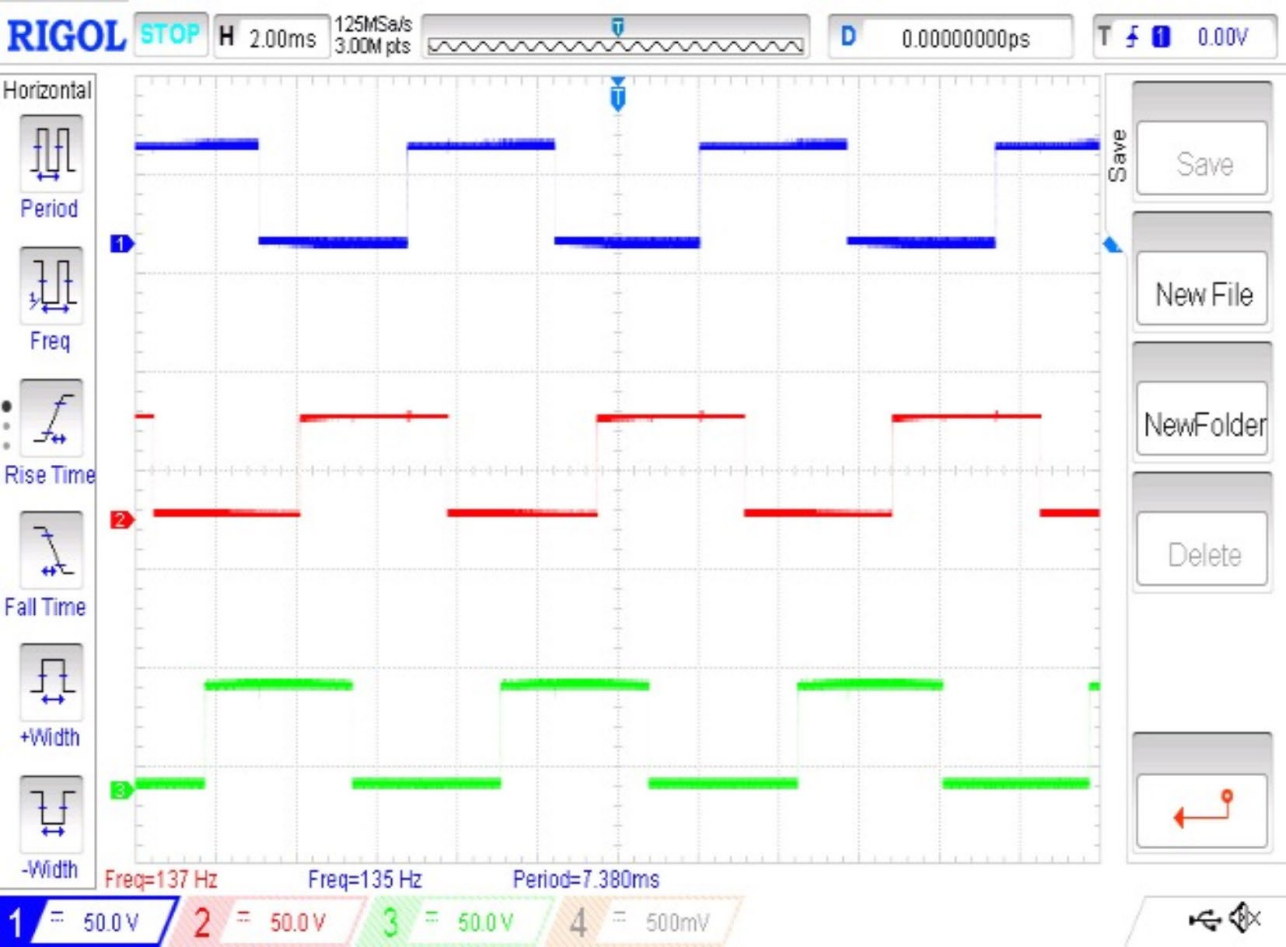
Hall sensor signals (x-axis 5 s/div, y-axis 5 V/div). For Fig. 7(a), (b) &(c)- (x-axis 1 s/div, y-axis 5 A/div).

The performance of a BLDC motor is evaluated based on its smooth starting under load conditions and its consistent input current. Figures 7 and 8 illustrate the stator current waveforms of the three phases and the controlled switching pulse generated by the conjunction of three Hall effect signals, respectively. These signals, generated by an integrated encoder based on the rotor’s position, are crucial for motor control.

**Fig. 9:**
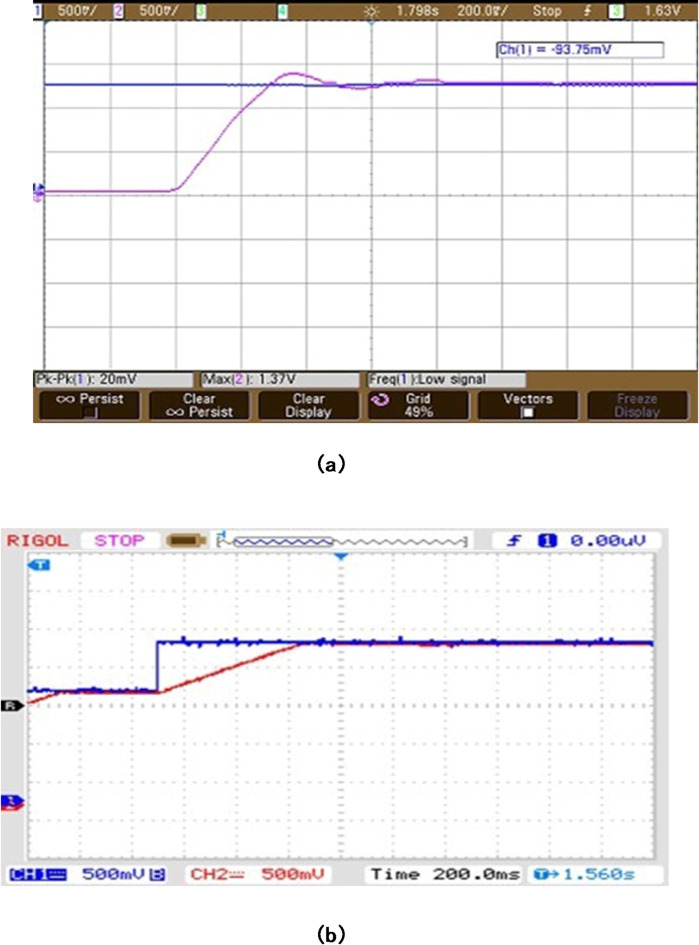
(**a**) Rated speed waveform at 2500 rpm (x-axis 1 s/div, y-axis 500 rpm/div). (**b**) Speed waveform at 1500 rpm (x-axis 0.02 s/div, y-axis 750 rpm/div).

The current waveforms shown in Fig. 7 (a), 7(b) and 7(c) go in concurrence with the simulation results. The current waveforms thus measured are within the limits and it ensures that the BLDC motor is very much capable of managing the temperature thereby providing much better reliability and longevity. It also signifies that the electromagnetic noise and mechanical vibrations are under control. From the hall sensor signal shown in Fig. 8, it is observed that each phase is clearly displaced by an angle of 120◦.It is observed that it provides a more precise rotor position control for appropriate commutation. Therefore it enhances the efficiency and performance of the entire system. Figure 9 (a) and 9(b) shows the speed waveforms of the BLDC moto under no load and loaded conditions respectively. It is observed that the speed in Fig. 9(a) for unloaded condition increases more gradually and settles down faster at 0.4 s and similarly for the curve shown in Fig. 9(b) under loaded condition the speed control is smooth and desirable. Moreover it settles at 0.8 s without any distortion, thereby proving that fuzzy logic controller is one of the best choice for the closed loop speed control operation of the BLDC motor.

**Fig. 10:**
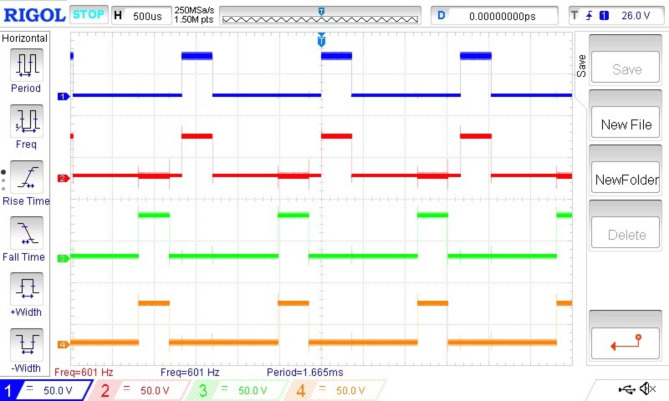
PWM gate pulses to VSI (x-axis 2.5 msec/div, y-axis 2 V/div).

It is found that the speed control thus obtained by using fuzzy logic controller is smooth, precise, ripple free, quick response time and stable. Additionally wide range of control ranging from 1500 rpm to 3000 rpm can be achieved, which makes the BLDC motor as one of the significant choices for Electric Vehicle applications. Figure 10 shows the gate pulses given using pulse width modulation. It ensures that the pulses are appropriate in such a way that the motor is capable of tracking the desired reference speed.

**Fig. 11:**
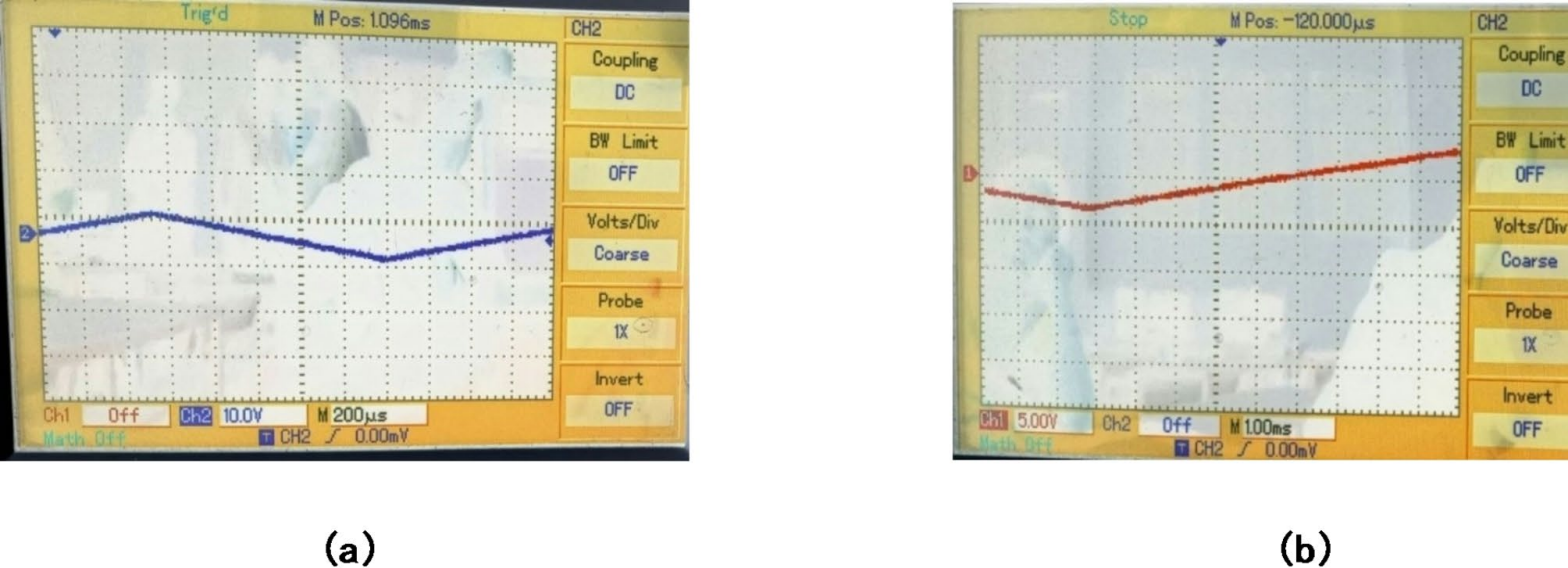
(**a**) Torque waveform of BLDC motor with PID controller and (**b**) Torque waveform of BLDC motor with FLC (x-axis 0.02 s/div, y-axis 1.01 Nm/div)).

The torque waveform for the BLDC motor under loaded conditions is shown in Fig. 11(a) & 11(b) respectively, emphasizing how torque pulsations are affected by both PID and fuzzy logic controllers. The Fuzzy Logic controller maintains a smooth and linear torque response with little noise or oscillations when a load is added at t = 0, increasing the initial torque to about 1.05 Nm. This suggests that the motor can manage abrupt load fluctuations with a faster recovery and stable performance thanks to the fuzzy logic controller’s ability to minimize torque ripple. On the other hand, there are noticeable oscillations in the torque waveform with the PID controller, which indicates greater torque pulsations. For electric vehicle applications that require steady and smooth torque production, these variations can lower operational smoothness, raise mechanical stress, and affect motor efficiency.

The comparison demonstrates that the fuzzy logic controller considerably lowers torque pulsations in contrast to the PID controller, resulting in a motor operation that is more reliable and efficient. This feature enhances overall smoothness and control, reduces vibration, and makes driving an electric vehicle simpler and more enjoyable. The ability of the fuzzy logic controller to sustain constant torque under a range of loads emphasizes further how well-suited it is for EV applications that demand high precision and little ripple.

**Fig. 12:**
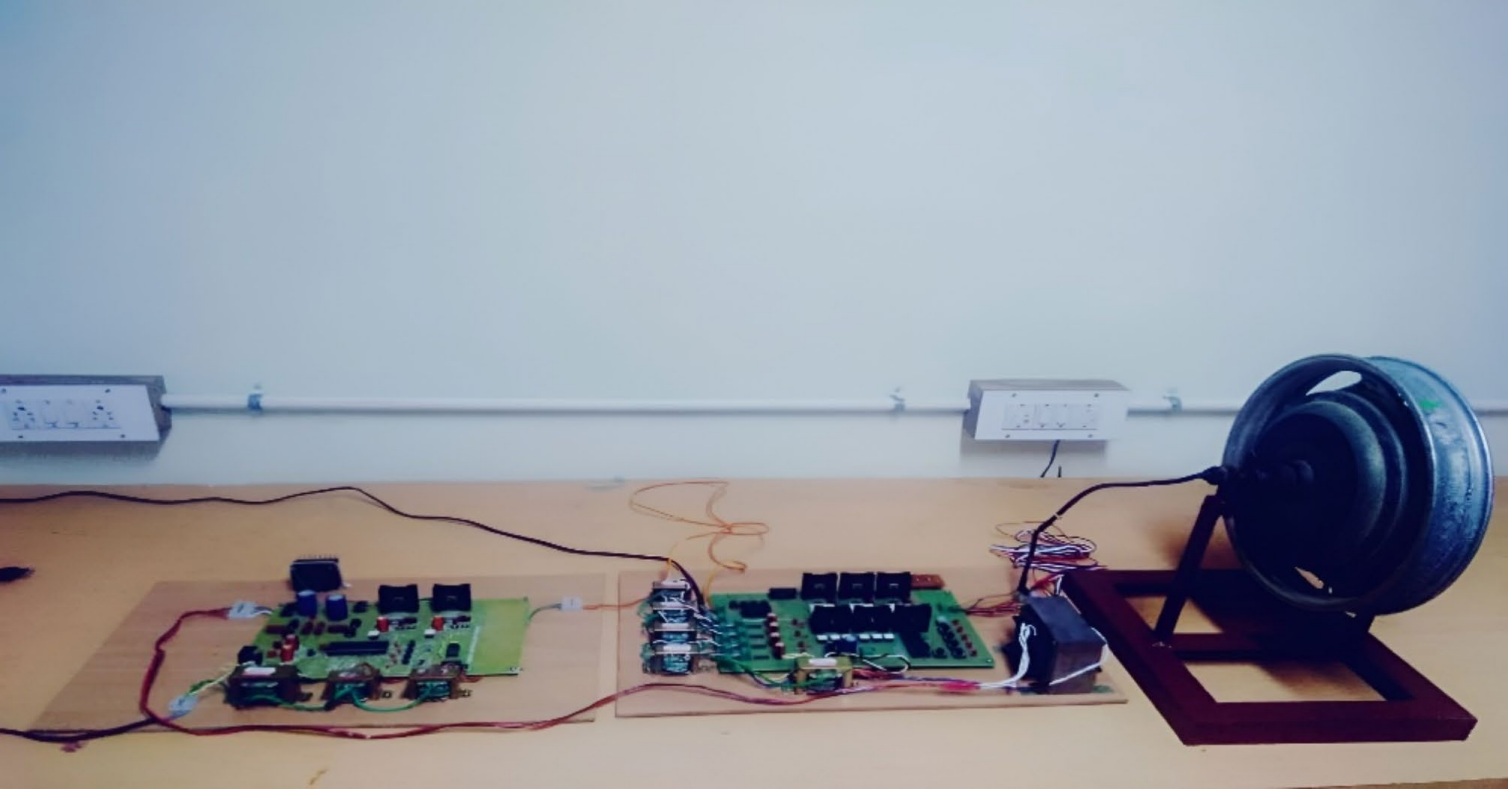
Experimental set up for speed control of BLDC motor.

Figure 12 shows the hardware set of BLDC motor speed control using fuzzy logic controller. The specifications of the BLDC motor used is as follows: BLDC Motor data (Experimental): stator phase resistance = 3.07 Ω stator phase inductance = 6.5 mH, Torque constant = 0.22 Nm/A, Po = 746 W; Io = 2.5, V = 310 V, *N* = 3000 rpm, *P* = 2.

In this setup, a DC generator is connected to the shaft of the BLDC motor to produce load variation. The BLDC motor powers the DC generator, which transforms the motor’s mechanical energy into electrical energy. A resistive load bank is subsequently connected to the generator’s electrical output. The resistance in the load bank can be changed to accurately manage the load on the DC generator and, in turn, the BLDC motor. When the resistance of the load bank is increased, the load on the generator increases thereby generating a higher torque demand on the BLDC motor and vice versa. With this technique, we can replicate actual operating circumstances in which the motor encounters abrupt or gradual variations in load.

A digital storage oscilloscope (DSO) and a power analyser were among the measurement tools used to record voltage, current, and speed waveforms in order to record and examine the motor’s response to these circumstances. To track real-time torque variations, a torque sensor was also used.Thus, the configuration makes it possible to thoroughly assess how the PID and fuzzy logic controllers handle torque pulsations, especially when there are dynamic load variations. Because a DSP-based system was used to create the controllers, real-time control and a rapid reaction to load variations were guaranteed. The Fuzzy Logic controller’s ability to reduce torque ripple and ensure quick recovery from disturbances is demonstrated by the smooth and stable torque waveform seen in Fig. 12, which offers a more dependable and smoother performance for electric vehicle applications. The BLDC motor was operated under various load situations (no-load, moderate-load, and full-load) during the testing process, and the effectiveness of both controllers was assessed during several test cycles. Ten seconds were allotted to each test, during which time data was recorded and examined.

In a nutshell this method of connecting a DC generator to a resistive load bank enables a realistic, regulated load variation, proving that the fuzzy logic controller is better at handling torque fluctuations than the PID controller.

## Handling real-time computational constraints in FLC for embedded EV motor controllers

This section discusses the real-time computational difficulties involved in integrating the Fuzzy Logic Controller (FLC) in embedded EV motor controllers in order to supplement the experimental results. Since the Embedded systems usually have limited resources, FLC’s computational efficiency must be maximised in order to achieve precise and timely motor control.

### Fuzzy rule base and membership functions optimisation

The number of fuzzy rules and membership functions are the crucial factors that affect the computational complexity of the FLC. The optimization of the FLC was implemented as follows:

(i) Simple Rule Base: To capture the key control dynamics and also to minimise the computational load the essential rules were carefully derived from the rule set.
(ii) Effective Membership Functions: Since the Gaussian and trapezoidal membership functions are more complex, Triangular membership functions were used to make the computations more simpler and easier.

By drastically lowering the amount of actions needed for each control cycle, these optimisations preserve control performance.

### Implementation of look-up table (LUT)

The precomputed fuzzy rules the response obtained were stored in the Look up table to improve the performance significantly. The above technique eliminated the the requirement of real-time rule evaluation and defuzzification thereby allowing quicker control response. The LUT offers several advantages including, (i) instantaneous rule evaluation which in turn reduces the delay in computation by retrieving the control outputs fast, (ii)lesser usage of memory while maintaining control responses in higher resolution.

### Optimization of the hardware

The TMS320C6713 DSP processor, which has a clock speed of 225 MHz and special resources for real-time motor control, was used to implement the FLC on a TMDSDSK6713 DSP Starter Kit.The significant optimization includes:

(i) Utilisation of the DSP’s parallel processing capabilities, fuzzification, rule evaluation and defuzzification are done simultaneously.
(ii) To ensure the minimal latency, the real time interrupts were given priority to complete the control tasks.
(iii) Fixed-point arithmetic was used in place of floating-point operations to save processing time and memory use.

### Simplified techniques for defuzzification

The weighted average method is used for the optimization of the defuzzification process.When compared with conventional centroid based methods, the weighted average method is computationally less intensive. This simplification guarantees the generation of control signals without sacrificing precision.

### Benchmarks and performance evaluation

The TMDSDSK6713 DSP Starter Kit was used to assess the proposed FLC’s real-time computational efficiency. The following constitute crucial performance indices:

(i) Computational time per control cycle: It is of the order of 0.02ms which is well within the acceptable time limit for the real time motor control.
(ii) Control Response Time: It is of the order of 0.05s which proves its capability in handling the dynamically varying load more efficiently.
(iii) Utilization of the Resources: Compared to a conventional FLC implementation without LUT optimisation, memory utilisation was lowered by 30%.

### Evaluation in comparison with PID controller

A comparative analysis of the computational performance of the FLC and a traditional PID controller was conducted. Despite using less computing power, the PID controller performed worse in terms of control in dynamic operating environments. The optimised FLC is a better option for EV motor control since it was able to strike a compromise between control accuracy and computational economy.

Through hardware-specific optimisations, rule-based optimisation, LUT implementation, and streamlined defuzzification approaches, the suggested FLC implementation successfully handles real-time computational limitations in embedded EV motor controllers. The results of the experiments performed on the TMDSDSK6713 DSP Starter Kit confirm that the optimised FLC provides greater control performance while satisfying the demanding real-time demands of EV applications. These results highlight FLC’s potential as a reliable and computationally effective solution for contemporary EV motor control systems.

## Limitation and future scope

### Limitation

It should be noted that parameter variations brought on by elements like temperature changes and motor ageing are not taken into consideration by the state-space model developed in this work. Over time, these impacts may affect the accuracy of the control system and cause variations in motor performance. Consequently, even while the model offers a strong foundation for comprehending the dynamics of the motor in optimal circumstances, more research into the addition of these variable components may improve the model’s applicability in the real world.

### Future scope

Hybrid control techniques may be investigated in the future to improve the accuracy and versatility of BLDC motor speed control. Fuzzy logic can be used in conjunction with more sophisticated methods such as model predictive control (MPC) or fractional-order PID (FOPID) to enhance dynamic responsiveness and disturbance rejection under various load scenarios. This hybrid technique is ideal for real-time EV applications because it guarantees more accurate speed regulation and robustness.

## Conclusion

This study investigates the closed-loop control of BLDC motor speed control using a fuzzy logic controller to improve the dynamic performance of the motor for EV applications. The BLDC motor is modelled using the state space technique. The results thus obtained using a fuzzy logic controller are compared against the conventional PID controller. It is observed that the motor performance with the fuzzy logic controller outperforms that of the PID controller regarding smooth and continuous speed control, energy efficiency, dynamic, reliability, and adaptability to load disturbances. The output thus obtained using a fuzzy logic controller demonstrated superior performance parameters such as lesser settling time, no overshoots or undershoots, and almost zero steady-state error.

In contrast, the motor with PID controller shows fluctuations as well as overshoots with longer settling time and shows some steady-state error. Above all the BLDC motor with fuzzy logic controller exhibits a smooth transition of speed for various load conditions thereby making the fuzzy logic controller the best choice for electric vehicle applications. The simulation results and hardware results are in concurrence with each other. Thus it is concluded that fuzzy logic-based speed control for BLDC motors is highly reliable, efficient with smooth speed control operation and stable irrespective of the load variations proving them the best choice for electric vehicles.

## Data Availability

The data used to support the findings of this study are included within the article.
